# Adherence therapy for adults with type 2 diabetes: a feasibility study of a randomized controlled trial

**DOI:** 10.1186/s40814-023-01294-2

**Published:** 2023-04-27

**Authors:** Fatimah Alenazi, Monica Peddle, Daniel Bressington, Moeber Mahzari, Richard Gray

**Affiliations:** 1 School of Nursing and Midwifery, La Trobe University, Melbourne, Victoria 3086 Australia; 2 Department of Public Health, College of Public Health and Health Informatics, Qassim University, AlBukayriyah, Kingdom of Saudi Arabia; 3 School of Nursing and Midwifery, Deakin University, Melbourne, Australia; 4 Faculty of Nursing, Chiang Mai University, 110/406 Inthawaroros Road, Sri Phum District, Chiang Mai, 50200 Thailand; 5 Faculty of Health, Charles Darwin University, Ellengowan Drive, Darwin, Northern Territory 0810 Australia; 6 College of Medicine, King Saud Bin Abdulaziz University for Health Sciences, Riyadh, Kingdom of Saudi Arabia; 7 Department of Medicine, Division of Endocrinology, Ministry of National Guard, Health Affairs, Riyadh, Kingdom of Saudi Arabia; 8 King Abdullah International Medical Research Center, Riyadh, Kingdom of Saudi Arabia

**Keywords:** Diabetes, Feasibility trial, Adherence, Compliance, Concordance, Adherence therapy

## Abstract

**Background:**

Adherence Therapy is a candidate intervention to promote consistent medication taking in people with type 2 diabetes. The aim of this study was to establish the feasibility of conducting a randomized controlled trial of adherence therapy in people with type 2 diabetes who were non-adherent with medication.

**Methods:**

The design is an open-label, single-center, randomized controlled feasibility trial. Participants were randomly allocated to receive either eight sessions of telephone-delivered adherence therapy or treatment as usual. Recruitment occurred during the COVID-19 pandemic. Outcome measures—adherence, beliefs about medication, and average blood glucose (sugar) levels (HbA1c)—were administered at baseline and after 8 weeks (TAU group) or at the completion of the treatment (AT group). Feasibility outcomes included the number of people approached to participate in the trial and the numbers that consented, completed study measures, finished treatment with adherence therapy, and dropped out of the trial. Fieldwork for this trial was conducted in the National Guard Hospital, a tertiary care provider, in the Kingdom of Saudi Arabia.

**Results:**

Seventy-eight people were screened, of which 47 met eligibility criteria and were invited to take part in the trial. Thirty-four people were excluded for various reasons. The remaining thirteen who consented to participate were enrolled in the trial and were randomized (AT, *n* = 7) (TAU, *n* = 6). Five (71%) of the seven participants in the adherence therapy arm completed treatment. Baseline measures were completed by all participants. Week 8 (post-treatment) measures were completed by eight (62%) participants. Dropout may have been linked to a poor understanding of what was involved in taking part in the trial.

**Conclusions:**

It may be feasible to conduct a full RCT of adherence therapy, but careful consideration should be given to developing effective recruitment strategies, consent procedures, rigorous field testing, and clear support materials.

**Trial registration:**

The trial was prospectively registered with the Australian New Zealand Clinical Trials Registry (ANZCTR), ACTRN12619000827134, on the 7th of June 2019.

**Supplementary Information:**

The online version contains supplementary material available at 10.1186/s40814-023-01294-2.

## Key messages regarding feasibility

- What uncertainties existed regarding the feasibility? It was unclear if the adherence therapy would be feasible in people with type 2 diabetes. However, we identify the possible challenges to conduct the intervention in the Middle Eastern context.
- What are the key feasibility findings? Participants in this trial, seemingly, did not have a good understanding of randomization particularly about the possibility of being allocated to a treatment as usual as a comparator group.
- What are the implications of the feasibility findings for the design of the main study?

The recruitment rate was lower than in studies in a similar population. This could be avoided by ensuring that potential participants have a clear understanding of random allocation, intervention, consent form, and the benefit of participating in the control group. Also, using RedCap as an online data record form was difficult as it does not support the Arabic language.

## Background

Type two diabetes is a common health problem affecting 9% of adults globally and is associated with poor health outcomes and high economic burden [1]. Health-related microvascular and macrovascular complications, including retinopathy, nephropathy, neuropathy, and cardiovascular diseases, are common consequences of diabetes and are a major cause of disease morbidity and mortality [2, 3]. The treatment of diabetes includes lifestyle modification and medication to maintain optimal blood glucose, prevent diabetes-related complications, and enhance the quality of life [4, 5]. Many people with type 2 diabetes find it challenging to take medication as prescribed [6–8] due to the complexity of treatment regimens [7], medication beliefs [9], and access to healthcare [10]. For example, the authors of a cohort study involving 200,000 people with diabetes in the USA observed that more than 30% of participants frequently missed medication [11]. In the Middle East, the prevalence estimates of adherence to diabetes medication are inconsistent, likely due to differences in sampling procedures and how adherence was measured [12, 13]. A review of 19 studies examined medication adherence in people with long-term conditions including diabetes in the Middle East and reported adherence rates ranging from 1 to 88% [14].

Authors of multiple systematic reviews have investigated factors related to poor adherence to diabetes medication [12, 15–20]. A systematic review of 21 studies coded reasons for non-adherence under three themes: (1) personal, (2) condition, and (3) healthcare and system [12]. Similar factors impacting adherence to diabetes medications were identified in a systematic review of observational studies conducted in the Middle East [16]. The combination of factors that impact medication adherence may suggest that interventions need to be person-centered and based on an individual assessment of experiences with medication [21].

There have been five systematic reviews [22–26] and two meta-analyses [26, 27] that examined the effectiveness of adherence interventions in diabetes. Reviews examined various types of adherence interventions including patient education [22, 23, 28], telephone follow-up [22, 23], in-person visits [26], short messaging service (SMS) reminders [23, 28], and decision making [26]. For example, Presley et al. [26], report a systematic review and meta-analysis involving 59 randomized controlled trials testing the effectiveness of adherence interventions. Fifty-seven trials were included in the meta-analysis. A large effect size (0.87) was reported against medication adherence and blood glucose levels. Included trials were rated as having a moderate or high risk of bias [26]. The review authors concluded that no intervention was obviously more effective than any other [23, 24, 29]. Most trials included in the reviews [26, 29] were conducted in the USA which may impact the generalizability of the findings particularly to other cultures and contexts. There is a scientific case for testing, within the context of well-designed RCTs, novel interventions to improve the consistency of medication taking in people with type 2 diabetes.

Adherence therapy (AT) [30] is a candidate novel intervention. AT is a manualized structured intervention based on motivational interviewing (exploring ambivalence about taking medication) and cognitive behavioral (problem-solving, challenging medication beliefs) techniques to improve medication adherence. The intervention has been tested in people experiencing mentally ill health, hypertension, and Parkinson’s disease. For example, a randomized controlled trial (RCT) by Alhalaiqa et al. [31] involving 136 patients with hypertension showed a 37% improvement in medication adherence in people receiving AT compared with treatment as usual (TAU). Seven sessions of AT were compared to TAU in 76 patients with Parkinson’s disease [32]. A significant improvement in medication adherence was reported between the groups with an odds ratio (OR) of 8.2 (95% CI 2.8, 24.3) [32]. There have been two systematic reviews of adherence therapy in people experiencing mentally ill health [33, 34]. Gray et al. reported a systematic review and meta-analysis of six randomized controlled trials testing adherence therapy in 725 patients with schizophrenia. AT was associated with a significant difference in psychiatric symptoms with a SMD (standardized mean difference) of 0.56 (small to medium effect size) [34]. A second systematic review including four randomized controlled trials evaluated the effectiveness of applying AT in people with schizophrenia and reported that AT was not effective in improving medication adherence [33]. In both reviews, the included trials were rated as having a low to moderate risk of bias [35, 36].

Adherence therapy has not been tested in people with type 2 diabetes in any context [37]. It is important to establish the feasibility of conducting a trial of AT to inform a main study. Testing AT in the Middle Eastern context is warranted because of the high number of adults with diabetes in the region. The Middle East and North Africa) the region has the second highest burden of diabetes globally with a prevalence of 13% (IDF, 2019). Further, there is some, albeit limited, evidence suggesting that there are specific issues related to treatment adherence within the Middle Eastern context [35].

### Aim

The aim of this study was to establish the feasibility of conducting a full randomized controlled trial of AT in people with type 2 diabetes in the Middle Eastern context.

The study protocol was prospectively registered before the first participant was recruited to the trial on the Australian New Zealand Clinical Trials Registry (ANZCTR), ACTRN12619000827134 registration DOI:https://anzctr.org.au/Trial/Registration/TrialReview.aspx?id=377588&isReview=true. A trial protocol has also been published [36].

## Methods

The reporting of this feasibility trial complies with the Consolidated Standards of Reporting Trials (CONSORT) guidelines extended for randomized pilot and feasibility trials [38] and the guidelines for reporting trial protocols and completed trials modified due to the COVID-19 pandemic and other extenuating circumstances (CONSERVE) [39].

### Trial design

The study design is a parallel group, open-labeled, randomized trial to determine the feasibility of AT compared to treatment as usual in people with type 2 diabetes.

### Participants

Participants were recruited from an outpatient endocrinology/diabetes clinic in the National Guard Hospital, Riyadh, Saudi Arabia. They included adults who had been diagnosed with type 2 diabetes for at least a year, were prescribed medication for the treatment and/or management of diabetes (e.g., oral hypoglycaemics or insulin injections) and were rated by healthcare workers as non-adherent to treatment (defined as a score lower than 25 on the Medication Adherence Reporting Scale (MARS-5)) (indicative of being non-adherent) [40]. Potential participants with conditions that may impair participation in the study, including terminal illness, brain injury, memory problems, and hearing deficits, were excluded. Those participating in other trials or treatments involving any psychological or psychosocial intervention were also not able to take part.

### Treatment as usual (TAU)

All participants continued to receive treatment as usual for the duration of the trial. Treatment as usual consisted of outpatient clinic appointments, diabetes medications, and patient education. The frequency of clinic appointments was based on individual patient needs and typically lasted 20 min. Clinic visits included medication review, health checks for diabetes complications, patient education, and referral to other clinical services as required. Patient education sessions were facilitated by an educator who provided didactic information about medication administration and more broadly about diabetes.

### Patient and public involvement

A cultural adaptation study—that included discussions about patients’ views and feedback on both adherence therapy and trial methodology—was conducted to inform the design of this feasibility study. The methodology of the cultural adaptation study has been described elsewhere in detail [36]. In summary, four adults with diabetes from the diabetes outpatient clinic in the National Guard Hospital agreed to participate in the cultural adaptation study and received adherence therapy. Participants were asked to share their views of the proposed treatment and trial design. During interviews, participants stated that they thought there needed to be at least one session discussing the importance of blood glucose monitoring. They also expected to be asked to provide a blood glucose reading at every session. Participants also reported that they found it easier to identify issues with medication taking by talking about a “typical day.” Regarding the trial methodology, patients reported that they did not like getting SMS (short messaging service) from the researcher and would rather information about the study was sent via a specific third-party messaging application that is extensively used in the Middle Eastern countries. Amendments to the study protocol were made, predominantly to the intervention, based on patient feedback. For example, each adherence therapy session started by asking patients about their most recent blood glucose reading. As part of the assessment phase of adherence therapy patients were asked to talk through a “typical day” with the educator who deliver the intervention. During the intervention, time was allocated to talking about the not-so-good and the good things about blood glucose monitoring. Finally, all communication between the research team and patients was undertaken using a third-party messaging application.

### Intervention (adherence therapy)

AT is described against the 12-item Template for Intervention Description and Replication Checklist (TIDieR) guidelines [41]. TIDieR guidelines have 12 items that include the brief name of the intervention, intervention description, intervention material, intervention procedures, intervention provider, mode of delivery, location of delivery, intervention period and frequency, intervention tailoring, planned intervention fidelity, and actual intervention fidelity.

1. Brief name. Adherence therapy in addition to TAU.
2. Why (intervention description). AT is a structured, pragmatic, patient-centered intervention combining elements from motivational interviewing and cognitive behavioral therapy. AT aims to enhance the consistency with which people take prescribed medications and is delivered by a trained health worker over eight individual sessions.
3. What (materials and procedures). AT is a manualized intervention. The manual can be accessed via this link: https://figshare.com/articles/online_resource/Adherence_therapy_manual/14298335. Templates to structure and guide individual sessions with patients are described in the manual. Each session is structured according to a ten-step procedure: (1) feelings check (e.g., “how are you doing today”), (2) reviewing the previous session and checking if homework has been completed, (3) checking if there are pressing issues that need to be discussed in the session, (4) setting an agenda and time limit for the session, (5) reminding the patient they can stop if they need a break or want to terminate the session, (6) completing the task for the session (e.g., exploring ambivalence), (7) summarizing the conversation, (8) agreeing homework, (9) planning the next session, and (10) feelings check at the end of the session.
4. Who provided the intervention? AT was delivered by a master’s prepared diabetes educator (FA), who completed online and face-to-face training in AT. The training lasted approximately 40 h.
5. How and where was the intervention delivered? The intervention was delivered to individual participants on a one-to-one basis over the telephone. Additionally, secure end-to-end encryption (third-party messaging service) reminders were sent a day before each session to confirm participant’s availability.
6. When and how much (intervention period and frequency). A total of eight sessions were administered over a maximum eleven-week period. Ideally, sessions were provided on a weekly basis. Each session lasted between 10 and 30 min.
7. Tailoring the intervention. The intervention was person-centered and adapted based on the adherence assessment completed during the first session. For example, if a participant was experiencing complex problems with medication and this was the key barrier to consistent medication taking then more time (and sessions) would be spent focusing on this element of the intervention.
8. Modifications. The study used a modified form of the original manual taking into consideration cultural acceptability. For example, we added an additional therapeutic technique—“tell us about a typical day”—to the intervention based on feedback from patients during the cultural adaptation phase of our work.
9. How well (planned). We aimed to audio record at least ten AT sessions that would be independently rated against the adherence therapy scale (a fidelity measure) to check that the intervention was being delivered faithfully.

### Control group

Participants in the control group received treatment as usual.

### Outcomes

#### Primary outcomes (feasibility)

The pre-specified primary outcomes of the trial were to establish the number of patients that are as follows:

- Were approached to participate and requested more information about the trial.
- Consented to be approached by the research team.
- Were invited to participate and consented to take part.
- Completed baseline measures.
- Completed treatment with adherence therapy (treatment completion was defined as attending five of the eight sessions within 11 weeks of treatment starting. A completed session was defined as engaging in the intervention for at least 10 min continuously).
- Completed all post-intervention outcome measures.

### Additional feasibility outcomes (amendments to the protocol)

Additional to the feasibility outcomes specified in the trial protocol, post hoc, we decided to examine the total number of AT sessions that were as follows:

- Planned
- Delivered
- Rescheduled by participants
- Rescheduled by the educator

We also examined the feasibility of collecting data on treatment fidelity by counting the total number of participants:

- That were asked if they would agree to an AT session being audio-recorded for fidelity assessment.
- That verbally agreed (checking of consent) for a session to be audio recorded for fidelity assessment.

Finally, we collected information on the number of times participants were asked to complete the follow-up assessment.

### Treatment fidelity

Treatment fidelity was determined by a researcher (MM) who was not involved in delivering the intervention using the AT checklist (supplementary document 1). The measure has been used in previous adherence therapy trials (Gray et al. 2005). The checklist is a seven-item measure rated on a 4-point scale. There is a descriptor for each item. A score of 12 of above is indicative of high fidelity to treatment.

### Secondary outcomes (measures)

Clinical outcome measures—blood glucose, medication adherence, and medication beliefs—were administered at baseline and post-intervention (8 weeks after the baseline assessment in the control group). All questionnaires used in this study have been translated and validated in Arabic [42].

### Self-reported blood glucose level

Measured by asking participants to self-report their most recent glycated hemoglobin (HbA1c) test results (a measure of the average blood glucose level in the last 3 months) that is part of the standard of care for patients attending the diabetes outpatient clinic. HbA1c levels were coded as normal in this study if the reported result was between 7 and 8% [8].

### Self-reported medication adherence

Medication adherence was determined using the Medication Adherence Reporting Scale (MARS-5) [40]. The MARS has five items scored on 1 (always) through 5 (never) scale. The MARS generates a total score ranging from 5 (frequently misses medication) to 25 (almost never misses medication). Chan et al. [40] demonstrated that the MARS scale as good psychometric properties.

### Beliefs about medication

Patient’s medication beliefs were measured using the 12-item Beliefs about Medication Questionnaire (BMQ) which is an extensively used attitudinal scale [43]. The measure produces a total score ranging from 12 to 60. The psychometric properties of the BMQ have been established in this population [42].

### Laboratory notes

Additionally, throughout the trial, the research team maintained detailed laboratory notes to document any unexpected issues that occurred during the conduct of the trial.

### Feasibility sample size

As we are reporting a feasibility study, a formal sample size calculation was not undertaken [44]. Authors have suggested that sample sizes of between 24 [45] and 50 [46] are required to provide meaningful information about the feasibility of conducting a full trial. In this trial, we aimed to recruit 40 participants.

### Randomization

We used a web-based independent service—Sealed Envelope (www.sealedenvelope.com) to undertake randomization. Sealed Envelope generates a random sequence in permuted blocks of four, six, or eight to ensure balanced groups [47]. The allocation ratio was 1:1. centralized external randomization services ensure allocation concealment.

Following obtaining written informed consent, the researcher (FA) created a participant case record form (CRF) and generated a unique study identification number. The participant identification number was entered into the study page on the sealed envelope website that generated group allocation. Group allocation was emailed to the study researcher (FA), who then sent a third-party messaging service to the participant informing them that they were in the AT or control group.

### Blinding

This was an open-label feasibility trial. Neither the researcher nor the participants were blind to group allocation. Blinding of researchers was not considered important in achieving the feasibility objectives of the research.

### Data collection

Patient-reported outcomes, including HbA1c, adherence, and beliefs, were collected at baseline –week 0—and at end of the study between weeks 9 and 11. Participants that did not complete questionaries at week nine were sent reminder messages after seven days (week 10) and—if they did not respond—after 14 days (week 11) asking them to complete the post-intervention measures. Data on the number of patients asked if they would like to take a part in the research, gave consent to be approached about the study by a researcher, and provide contact information were collected by clinicians working in the diabetes clinic (the treating team). Once formal consent was obtained, participants were sent a second link—also using a third-party messaging service—to the baseline assessment. All assessments were completed online using REDCap which is an online electronic case record form.

### Data analysis

Data were initially downloaded from REDCap as Excel XLSX files. Data were checked and cleaned using the procedures described in the trial protocol [36] and then imported into SPSS (version 25) for analysis. Descriptive statistics (mean, standard deviation, and proportions) were used to describe the demographic and clinical characteristics of participants and feasibility outcomes.

### Ethics

The trial was approved by the King Abdullah International Medical Research Centre (KAIMRC) (reference number: RYD-19–419,812-147,869) and La Trobe University Human Research Ethics Committee (Reference number: HEC19221). All investigators completed Good Clinical Practice (GCP) training prior to the start of the trial. The trial was conducted according to the GCP standards.

### Consent procedure

The treating team provided verbal information about the trial to patients attending the diabetes outpatient clinic. Patients that expressed an interest in the trial were asked if they would consent to their contact information being shared with the researcher (FA) who would telephone them to explain the study in more detail. If, following the telephone conversation, patients indicated they were happy to take part in the trial, they were sent a link to the participant information form and e-consent procedure using REDCap. Participants were given two days to consider if they wanted to participate in the trial.

### Harms reporting

Adverse events that occurred during the conduct of the trial were recorded as per GCP requirements. Participants were asked to report any adverse events that occurred—specifically any medical issues and/or hospitalizations—to the researchers as soon as possible. Participants were also asked to report if any adverse events occurred at the final assessment. Harms reported by participants were recorded on a standard adverse event reporting form by the researcher and reported to the relevant ethics and trial steering committee.

### Amendments to the study protocol

This study was conducted during the COVID-19 pandemic; consequently, there were methodological modifications that needed to be made to the study protocol. Amendments are described below following the CONSERVE (41) reporting guidelines:

### Extenuating circumstances

The main aim of feasibility and pilot studies is to assess the possibility of conducting a full and appropriately powered clinical trial with respect to various aspects, such as recruitment and dropout rates [48]. As per our protocol, recruitment was intended to take place in person in the diabetes clinic. The COVID-19 pandemic resulted in lockdown restrictions before the start of recruitment which meant the proposed recruitment procedures were no longer appropriate.

### Important modifications

In response to the pandemic, we made three amendments to the protocol of our feasibility trial that were reviewed and approved by the relevant ethics committee.

1. Telephone recruitment

During the pandemic, routine clinical appointments were delivered over the telephone. We asked the treating clinical team if they would, during telephone consultations, explain the trial and ask if patients were interested in participating.

2. E-consent

We switched to using an electronic rather than paper-based consent procedure.

3. Sample size

The start of the trial was delayed by four months because of COVID-19 restrictions which meant that we had a shorter time to recruit participants. We anticipated this delay would impact our ability to recruit the target number of participants. We considered the ethical implications of conducting a trial where we were not confident we could recruit the required number of participants. Our decision to continue with the trial was informed by the knowledge that feasibility trials do not have specific sample size requirements and that important information to inform a full trial would still be generated from a smaller sample size [49].

### Responsible parties

The above modifications, including using the telephone for participant recruitment and electronic consent form, were initiated by the research team and submitted as a protocol amendment to the La Trobe University (LTU) Human Ethics Committee and King Abdallah Medical Research Centre (KAMRC) for review and approval. The National Guard Hospital also approved using telephone recruitment procedures. Finally, the trial steering committee was informed about and approved the amendments.

### Interim data

The modifications to the protocol were made prior to the first participant being recruited. Consequently, we did not need to (and were not able to) undertake any interim data analysis to inform our amendments.

## Results

### Trial feasibility

Care should be taken when interpreting the results of this trial due to the small number of participants.

### Recruitment

**Fig. 1:**
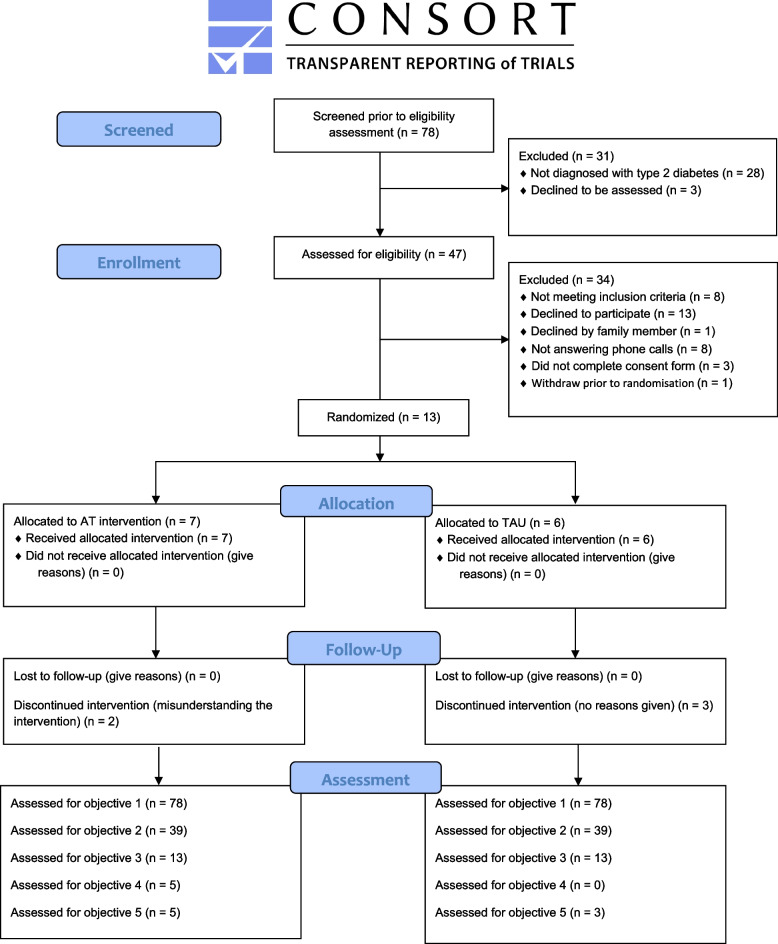
CONSORT flow

Figure 1 shows the flow of participants through the trial. The treating clinical team reported that they approached 78 patients about their interest in participating in the trial during routine telephone clinical consultations. Of these, the treating clinical team excluded 31 (39%) people, of which three declined to take a part in the study and twenty-eight did not meet the trial inclusion criteria. Forty-seven people were contacted by the trial researcher, of which thirteen provided written informed consent. All consenting participants completed baseline measures and were randomized. The recruitment process started on July 1, 2020. People who met trial eligibility criteria and who declined to participate were not asked the reason why they did not want to take part in the study because the ethics committee would not approve of collecting data from people that had not given consent. However, we note that people who declined to participate generally did so because they indicated that they were too busy or were not comfortable taking part in an intervention study.

### Drop out during the trial

Five participants (38%)—two in the AT group and three in the TAU group—dropped out of the study. The pattern of dropouts differed between the two groups. In the AT group, one participant dropped out after a single session of AT and one dropped out after two sessions. Both participants stated that they did not understand that they were participating in a trial and were receiving an experimental treatment. Both participants indicated that they thought they were being offered an extension of usual care. All three participants who dropped out from the TAU group did so at the point of randomization (when the researcher telephoned them to inform them of their group allocation) indicating that they no longer wished to participate in the study.

### Treatment completion and fidelity monitoring

Five (71%) of the seven participants randomized to AT met the criteria for treatment completion. Participants that completed treatment attended all the eight AT sessions. In total, 43 sessions of AT were delivered. Twenty-five (58%) sessions were delivered at the date/time agreed by the researcher. Patients asked to change the date or time of 18 sessions, eight of which were changed multiple times. No sessions were rescheduled by the researcher.

All the participants that completed more than one AT session (*n* = 6) were asked if they would consent to an AT session being audio recorded to check treatment fidelity. Three (50%) agreed to be recorded. The mean score on the AT checklist was 23.6 (sd = 6.1). All three scored greater than 12, indicating that AT was being delivered with a high degree of fidelity.

### Completion of outcome measures

Follow-up (end of treatment) measures were completed by eight (61%) participants. Five participants at the baseline and three participants at follow-up reported invalid HbA1c value (i.e., far outside the possible range). On average patients needed to be contacted twice (range 1 through 3) to successfully complete outcome measures.

### Use of electronic case record forms

We noted multiple issues with the use of electronic case record forms. One participant completed the baseline assessment four times. Four participants messaged the researcher to check if the assessment was received following the completion of the baseline measures.

#### Demographic characteristics of the participants

Table 1 shows the baseline demographic characteristics of participants. Participants were in their mid-40 s, two thirds identified as female, all but one participant was married, and all had children. Almost half of the patients had completed high school education and the majority were unemployed. Most participants had an annual family income of less than US $19,200.

**Table 1:** Baseline demographic characteristics of the participants

Characteristics	AT group *N* = 7	TAU group *N* = 6	All participants *N* = 13
Age in years			
*Median*	52	42.5	43
*Range*	37–60	32–60	32–60
Gender, *n* (%)			
*Female*	7 (100)	2 (33)	9 (69)
*Male*	0 (0)	4 (67)	4 (31)
Marital status, *n* (%)			
*Married*	6 (86)	5 (83)	11 (85)
*Widowed*	1 (14)	0 (0)	1 (8)
*Divorced*	0 (0)	1 (16.7)	1 (8)
Educational level, *n* (%)			
*None*	1 (14)	1 (17)	2 (15)
*High school*	1 (14)	4 (67)	5 (39)
*Secondary*	3 (43)	0 (0)	3 (23)
*Elementary*	2 (29)	0 (0)	2 (15)
*Diploma*	0 (0)	1 (17)	1 (8)
Occupation, *n* (%)			
*Employed*	0 (0)	3 (50)	3 (23)
*Unemployed*	7 (100)	3 (50)	10 (77)
Annual family income, USD			
*0–19,200*	7 (100)	4 (67)	11 (85)
*19,201–38,400*	0 (0)	2 (33)	2 (15)
Country of birth, *n* (%)			
*Saudi Arabia*		6 (100)	13 (100)
*Other*		0 (0)	0 (0)
Place of residence, *n* (%)			
*Lived in Riyadh*	6 (86)	4 (67)	10 (77)
*Lived outside Riyadh*	1 (14)	2 (33)	3 (23)
Presence of children*, n (%)*	7 (100)	6 (100)	13 (100)
*Median*	5	3	5
*Range*	5–9	2–9	2–9

### Clinical characteristics of the participants

#### Baseline measures

Table 2 shows the clinical characteristics of included participants. Across all study measures the only missing data were for self-reported HbA1c (*n* = 6; AT group, *n* = 3, TAU, *n* = 3). Seven (54%) participants (AT group = 4 and TAU = 3) reported their HbA1c results. Just over half of the participants self-reported normal HbA1c levels. The baseline MARS and BMQ scores were obtained from all the participants, and they were similar for the two groups suggesting patients were non-adherent and had negative attitudes towards treatment. Around two thirds of participants reported having one or more of eight comorbid conditions (hypertension, heart disease, high cholesterol, diabetic foot, glaucoma, kidney failure, hypothyroidism, and asthma). Hypertension was the most common comorbid condition reported in four patients followed by heart disease and high cholesterol in two patients each.

**Table 2:** Baseline clinical characteristics of the participants

Characteristics	AT group *N* = 7	TAU group *N* = 6	All participants *N* = 13
Duration of diabetes in years			
*Median*	5	3	5
*Range*	2–20	2–13	2–20
HbA1c, *n* (%)	*n* = 4	*n* = 3	*n* = 7
*Normal*	2 (50)	2 (67)	4 (57)
*Abnormal*	2 (50)	1 (33)	3 (43)
Medication measures, mean (SD)	*n* = 7	*n* = 6	*n* = 13
*MARS-5*	21 (3.00)	21.5 (2.66)	21.23 (2.74)
*BMQ-General*	41.14 (4.41)	40.83 (6.18)	41.00 (5.07)
Diabetes complications, *n* (%)	*n* = 7	*n* = 6	*n* = 13
*Yes*	0 (0)	1 (17)	1 (8)
*No*	7 (100)	5 (83)	12 (92)
Comorbidity, *n* (%)			
*Yes*	5 (71)	3 (50)	8 (62)
*No*	2 (29)	3 (50)	5 (38)

#### Differential drop out

Table 3 shows the demographic profile (baseline characteristics) of participants who completed and dropped out of the trial. Seemingly there were no important differences between the groups.

**Table 3:** Comparison of baseline demographic characteristics of study completers and dropouts

Characteristics	Completers (*n* = 8)	Drop-outs (*n* = 5)
Age in years		
*Median*	41	43
*Range*	32–60	40–60
Gender, *n* (%)		
*Female*	6 (75)	3 (60)
*Male*	2 (25)	2 (40)
Marital status, *n* (%)		
*Married*	8 (100)	3 (60)
*Widowed*	0 (0)	1 (20)
*Divorced*	0 (0)	1 (20)
Educational level, *n* (%)		
*None*	0 (0)	2 (40)
*High school*	2 (25)	3 (60)
*Secondary*	3 (38)	0 (0)
*Elementary*	2 (25)	0 (0)
*Diploma*	1 (12)	1 (33)
Occupation, *n* (%)		
*Employed*	2 (25)	1 (20)
*Unemployed*	6 (75)	4 (80)
Annual family income, USD		
*0–19,200*	7 (88)	4 (80)
*19,201–38,400*	1 (12)	1 (20)
Country of origin, *n* (%)		
*Saudi Arabia*	8 (100)	5 (100)
*Other*	0 (0)	0 (0)
Place of residence, n (%)		
*Lived in Riyadh*	8 (100)	2 (40)
*Lived outside Riyadh*	0 (0)	3 (60)
Presence of children*, n (%)*	8 (100)	5 (100)
*Median*	5	5
*Range*	3–9	2–7

#### Follow-up measures

Table 4 shows scores for outcome measures at follow-up. No inferential analysis was undertaken.

**Table 4:** Follow-up clinical characteristics

Characteristics	AT group *N* = 5	TAU group *N* = 3	All participants *N* = 8
Duration of diabetes in years			
*Median*	6	3	5.5
*Range*	2–20	2–12	2–20
HbA1c, *n* (%)	*n* = 2	*n* = 2	*n* = 4
Normal	1 (50)	2 (100)	3 (75)
Abnormal	1 (50)	0 (0)	1 (25)
Medication measures, Mean (SD)	*n* = 5	*n* = 3	*n* = 8
MARS-5	19.60 (3.91)	22.33 (0.58)	20.63 (3.29)
BMQ-General	37.20 (6.87)	39.33 (4.62)	38.00 (5.86)
Diabetes complications, *n* (%)	*n* = 5	*n* = 3	*n* = 8
Yes	0 (0)	1 (33)	1 (13)
No	5 (100)	2 (67)	12 (87)
Comorbidity, *n* (%)			
Yes	4 (80)	2 (67)	6 (75)
No	1 (20)	1 (33)	2 (25)

#### Harms

Three study participants reported adverse events during the trial, all in the AT group. There were two admissions to the hospital (serious adverse events) one because of high blood pressure, the second because of hyperglycemia that resulted in an admission to the emergency department. Finally, one participant reported they had tested positive for COVID-19 (adverse event). Reported harms were reviewed by a medical practitioner who did not consider that they were associated with the study intervention. The higher number of adverse events reported in the AT group could be explained by the increased contact between researchers and study participants in this arm of the trial. Harms were reported to the La Trobe University and KAMRC human research ethics committee and to the trial steering committee in accordance with GCP.

## Discussion

### Trial feasibility

The aim of this study was to test the feasibility of conducting a full-scale trial of adherence therapy in patients with type 2 diabetes in the Middle Eastern context. In this trial, we needed to ask 3 patients to get one to consent to participate in the trial, that is seemingly higher than in similar trials in this population. Typically, in adherence trials in people with diabetes, the consent rate is higher than we report [50–53]. For example, in a trial of an integrated care intervention to improve medication taking, the author invited 259 patients with diabetes to participate of which 182 consented and were randomized, that is to say, 1.4 patients needed to be asked to get one to consent [54]. We found only one previous feasibility trial of an adherence intervention (in this case a smartphone application) where recruitment rates were similar to our trial [55]. In the Huang et. al. trial, the authors needed to invite 3.4 people with diabetes to take part to get one to consent [55]. There are three possible explanations for our observation: first, in many trials testing adherence interventions diabetes, researchers tend to include patients that are good at sticking with their medication as well as those who regularly miss doses [52, 53, 55, 56].

Researchers may tend to avoid specifying non-adherence as an inclusion criterion to ensure they can recruit to target; however, this may create a ceiling effect because researchers are testing an intervention on people who are adherent to treatment. In our trial, we only included participants that met pre-specified criteria for being non-adherent with their medication (MARS score < 25), where one might logically expect patients who regularly miss doses of medication to be more hesitant about participating in a trial. Also, fieldwork for this trial was conducted during the COVID-19 pandemic whilst lockdown restrictions were in place, potentially impacting recruitment and ability to attain the target sample size. By necessity, recruitment was undertaken using telephone and text messaging and not using more traditional face-to-face processes where informal time can be given to discussing and explaining the trial with potential participants. Diabetes trials conducted during the pandemic did not, however, seem to struggle to recruit to time and target. For example, a trial of psychological intervention in 91 patients with diabetes managed to recruit 100% of the required sample [57].

### Intervention delivery

Almost 1 in 2 adherence therapy sessions were rescheduled by participants. Feasibility trials of psychosocial interventions in diabetes have not previously reported details of how often sessions with patients needed to be changed. These data are important because it impacts on treatment fidelity and on the cost and complexity of treatment delivery in a real-world clinical setting.

### Dropout

We observed apparent differential reasons for dropout between the two trial arms. Participants in the AT group seemingly did not fully understand they were consenting to take part in a clinical trial, rather they thought that they were receiving extended usual care. In the TAU group, participants dropped out because they did not understand the purpose or see the benefit of a comparator group where there was no active intervention. It is concerning, but perhaps not uncommon, for participants in clinical research to not understand the trial in which they are participating in despite researchers strictly following the consent processes reviewed and approved by relevant ethics committees. For example, a systematic review and meta-analysis of 103 trials report that a quarter of participants did not understand the purpose of a clinical trial, half did not understand randomization and almost a third could not explain the aim of the trial [58]. The apparent, poor understanding of the trial by study participants may be explained by our use of an electronic consent process. In a full trial, researchers need to pay close attention to ensuring that participants have a good understanding of the risks and benefits of the trials that they are consenting to participate in. Participants in the TAU group indicated when they were interviewed that they were expecting to receive an additional benefit (additional treatment) from taking part in the trial. It may be that in a definitive trial, a waiting list design is adopted so that all participants receive the AT intervention.

### Feasibility of an online data collection procedure

Using REDcap as a data, record form was challenging as it does not support Arabic as a core language. Specifically, it was difficult to format the text so that it could be read right to left. Also, after completing study measures an automatic message in English was shown on the screen indicating that all questions had been completed, this confused many participants who could not read English. We consider that these are important technical considerations that researchers conducting field work in the Middle Eastern context should consider when planning their studies.

We asked participants to self-report their most recent HbA1c test scores. Because HbA1c was not the primary outcome, it was considered pragmatic to collect this measure using self-report. In our trial the poor self-reporting, we observed may be explained by participants forgetting their results, having a poor understanding of HbA1c, or unidentified transcultural issues (assumed medical authority). Trivedi et al. investigated the validity of self-report as a method of measuring HbA1c [59]. Comparing self-report with laboratory results in a study involving 7597, the authors reported that 78% of patients were accurate in self-reporting HbA1c levels [59]. Although apparently a valid approach to measuring HbA1C, our observations from this trial suggest that self-report may not be feasible in adherence studies in the Middle Eastern context.

### Harms

Adherence therapy has been tested in 11 clinical trials to evaluate the effectiveness and safety of the intervention with most studies investigating it in people with psychiatric disorders. Only one trial by Maneesakorn et al. [60] reported any adverse events. However, these outcomes were due to accidents not related to the study. Other AT trials reported no adverse events or harms. The reporting of harms in adherence interventions in diabetes trials more generally is poor.

### Study limitations

In this feasibility trial, we identified several important limitations that are important to note. First, we did not test procedures for blinding participants or the researchers to group allocation. Consequently, we cannot comment on the feasibility or impact of blinding in future trials of adherence therapy. The researcher (FA) delivering the intervention also consented participants into the trial. It is plausible that this created some pressure on participants in the adherence therapy group to remain in the trial even though they wished to drop out—a type of social desirability bias (people stayed in the trial to help the researcher). There is little evidence to support this argument as dropout rates in our trial were higher than in similar trials.

We did not collect data on health economic outcome measures, number of TAU visits, and number of medications prescribed. Another limitation is that we did not meet the requirements of our sample size estimate, potentially limiting the generalisability of our observations. We also did not specify in the trial protocol the maximum number of weeks to complete adherence therapy, and consequently, there was the potential that participants perceived pressure to complete treatment within a time period that we had not specified. Additionally, we did not apply a priori stopping criteria in our trial. Finally, we have not used a specific procedure for monitoring adverse events in both groups, but we asked them at the last eight weeks (TAU) or at the completion of treatment (AT) if they have had any adverse events.

## Conclusions

It may be feasible to undertake a full trial of AT trial in people with type 2 diabetes that frequently miss doses of medication in the Middle Eastern population. That said there were important challenges in conducting our feasibility trial that should inform the development of a full-trial protocol. Due to the challenges of conducting this study, we recommend researchers to conduct an internal pilot to re-test some of the feasibility aspects before conducting a full RCT. Specifically, research groups should consider effective recruitment strategies (with COVID-19 contingencies), and consent and reconsent procedures to ensure that patients are providing true informed consent, rigorous field testing, and providing clear support materials to participants about how to complete online case record forms with a goal of improving the sample size.

## Supplementary Information

**Additional file 1.** CONSORT 2010 checklist of information to include when reporting a randomised trial*.

## Data Availability

The dataset supporting the conclusions of this article is available in the La Trobe repository, https://opal.latrobe.edu.au/account/home.

## Abbreviations

AT: Adherence therapy
ANZCTR: Australian New Zealand Clinical Trials Registry
RCTs: Randomized controlled trials
CONSORT: Consolidated Standards of Reporting Trials
CONSERVE: Guidelines for reporting trial protocols and completed trials modified due to the COVID-19 pandemic and other extenuating circumstances
MARS-5: Medication Adherence Reporting Scale
BMQ: Beliefs about Medication Questionnaire
TIDieR: Intervention Description and Replication Checklist TIDieR guidelines
TAU: Treatment as usual
HbA1c: Glycated hemoglobin
KAIMRC: King Abdullah International Medical Research Center
GCP: Good Clinical Practice

## References

[1] International Diabetes Federation. IDF Diabetes Atlas, 9th edn. Brussels, Belgium; 2019. Available from: https://www.diabetesatlas.org

[2] Deshpande AD, Harris-Hayes M, Schootman M (2008). Epidemiology of diabetes and diabetes-related complications. Phys Ther.

[3] Raghavan S, Vassy JL, Ho Y-L, Song RJ, Gagnon DR, Cho K (2019). Diabetes mellitus-related all-cause and cardiovascular mortality in a national cohort of adults. J Am Heart Assoc.

[4] Simon GE, Katon WJ, Lin EHB, Ludman E, VonKorff M, Ciechanowski P (2005). Diabetes complications and depression as predictors of health service costs. Gen Hosp Psychiatry.

[5] American Diabetes Association. Standards of medical care in diabetes. Dibetes Care. 2017;40:10

[6] Casagrande SS, Fradkin JE, Saydah SH, Rust KF, Cowie CC (2013). The prevalence of meeting A1C, blood pressure, and LDL goals among people with diabetes, 1988–2010. Diabetes Care.

[7] Coleman CI, Limone B, Sobieraj DM, Lee S, Roberts MS, Kaur R (2012). Dosing frequency and medication adherence in chronic disease. J Manag Care Pharm.

[8] American Diabetes Association (2019). 6. Glycemic targets: standards of medical care in diabetes—2019. Diabetes Care.

[9] de Vries ST, Keers JC, Visser R, de Zeeuw D, Haaijer-Ruskamp FM, Voorham J (2014). Medication beliefs, treatment complexity, and non-adherence to different drug classes in patients with type 2 diabetes. J Psychosom Res.

[10] Ong SE, Koh JJK, Toh SAES, Chia KS, Balabanova D, McKee M (2018). Assessing the influence of health systems on type 2 diabetes mellitus awareness, treatment, adherence, and control: a systematic review. PLoS One.

[11] Kirkman MS, Rowan-Martin MT, Levin R, Fonseca VA, Schmittdiel JA, Herman WH (2015). Determinants of adherence to diabetes medications: findings from a large pharmacy claims database. Diabetes Care.

[12] Krass I, Schieback P, Dhippayom T (2015). Adherence to diabetes medication: a systematic review. Diabet Med J Br Diabet Assoc.

[13] Cramer JA, Benedict A, Muszbek N, Keskinaslan A, Khan ZM (2008). The significance of compliance and persistence in the treatment of diabetes, hypertension and dyslipidaemia: a review. Int J Clin Pract.

[14] Al Qasem A, Smith F, Clifford S (2011). Adherence to medication among chronic patients in Middle Eastern countries: review of studies. East Mediterr Health.

[15] Peeters B, Tongelen IV, Boussery K, Mehuys E, Remon JP, Willems S (2011). Factors associated with medication adherence to oral hypoglycaemic agents in different ethnic groups suffering from type 2 diabetes: a systematic literature review and suggestions for further research. Diabet Med.

[16] Jaam M, Awaisu A, Ibrahim MI, Kheir N (2017). Synthesizing and appraising the quality of the evidence on factors associated with medication adherence in diabetes: a systematic review of systematic reviews. Value Health Reg Issues.

[17] Gellad WF, Grenard JL, Marcum ZA (2011). A systematic review of barriers to medication adherence in the elderly: looking beyond cost and regimen complexity. Am J Geriatr Pharmacother.

[18] Yap AF, Thirumoorthy T, Kwan YH (2016). Systematic review of the barriers affecting medication adherence in older adults. Geriatr Gerontol Int.

[19] Capoccia K, Odegard PS, Letassy N (2016). Medication adherence with diabetes medication: a systematic review of the literature. Diabetes Educ.

[20] Odegard PS, Capoccia K (2007). Medication taking and diabetes: a systematic review of the literature. Diabetes Educ.

[21] Brown MT, Bussell JK (2011). Medication adherence: WHO cares?. Mayo Clin Proc.

[22] Asante E (2013). Interventions to promote treatment adherence in type 2 diabetes mellitus. Br J Community Nurs.

[23] Sapkota S, Brien J-AE, Greenfield JR, Aslani P (2015). A systematic review of interventions addressing adherence to anti-diabetic medications in patients with type 2 diabetes–components of interventions. PloS One.

[24] Williams JLS, Walker RJ, Smalls BL, Campbell JA, Egede LE (2014). Effective interventions to improve medication adherence in type 2 diabetes: a systematic review. Diabetes Manag Lond Engl.

[25] Vermeire EI, Wens J, Royen PV, Biot Y, Hearnshaw H, Lindenmeyer A. Interventions for improving adherence to treatment recommendations in people with type 2 diabetes mellitus. Cochrane Database Syst Rev. John Wiley & Sons, Ltd; 2005.Cited 2020 Apr 24; Available from: 10.1002/14651858.CD003638.pub2/full 10.1002/14651858.CD003638.pub2 PMC9022438 15846672

[26] Presley B, Groot W, Pavlova M (2019). Pharmacy-led interventions to improve medication adherence among adults with diabetes: a systematic review and meta-analysis. Res Soc Adm Pharm RSAP.

[27] Alshehri AA, Jalal Z, Cheema E, Haque MS, Jenkins D, Yahyouche A (2020). Impact of the pharmacist-led intervention on the control of medical cardiovascular risk factors for the primary prevention of cardiovascular disease in general practice: a systematic review and meta-analysis of randomised controlled trials. Br J Clin Pharmacol.

[28] Kini V, Ho PM (2018). Interventions to improve medication adherence: a review. JAMA.

[29] Ismail K, Winkley K, Rabe-Hesketh S (2004). Systematic review and meta-analysis of randomised controlled trials of psychological interventions to improve glycaemic control in patients with type 2 diabetes. Lancet.

[30] Gray R, Leese M, Bindman J, Becker T, Burti L, David A (2006). Adherence therapy for people with schizophrenia: European multicentre randomised controlled trial. Br J Psychiatry.

[31] Alhalaiqa F, Deane KHO, Nawafleh AH, Clark A, Gray R (2012). Adherence therapy for medication non-compliant patients with hypertension: a randomised controlled trial. J Hum Hypertens.

[32] Daley DJ, Deane KHO, Gray RJ, Clark AB, Pfeil M, Sabanathan K (2014). Adherence therapy improves medication adherence and quality of life in people with Parkinson’s disease: a randomised controlled trial. Int J Clin Pract.

[33] Hegedüs A, Kozel B (2014). Does adherence therapy improve medication adherence among patients with schizophrenia? A systematic review. Int J Ment Health Nurs.

[34] Gray R, Bressington D, Ivanecka A, Hardy S, Jones M, Schulz M, et al. Is adherence therapy an effective adjunct treatment for patients with schizophrenia spectrum disorders? A systematic review and meta-analysis. BMC Psychiatry. 2016;16. Cited 2020 Apr 26. Available from: https://www.ncbi.nlm.nih.gov/pmc/articles/PMC4822226/10.1186/s12888-016-0801-1 PMC4822226 27048373

[35] Alsairafi ZK, Taylor KMG, Smith FJ, Alattar AT (2016). Patients’ management of type 2 diabetes in Middle Eastern countries: review of studies. Patient Prefer Adherence.

[36] Alenazi F, Peddle M, Bressington D, Mahzari M, Gray R (2021). A study protocol for a feasibility trial of telephone-delivered adherence therapy for adults with type 2 diabetes. Nurs Open.

[37] Alenazi F, Bressington D, Shrestha M, Peddle M, Gray R (2021). Effectiveness of adherence therapy in adults with type 2 diabetes: a systematic review. Int J Environ Res Public Health.

[38] Eldridge SM, Chan CL, Campbell MJ, Bond CM, Hopewell S, Thabane L (2016). CONSORT 2010 statement: extension to randomised pilot and feasibility trials. BMJ.

[39] Orkin AM, Gill PJ, Ghersi D, Campbell L, Sugarman J, Emsley R (2021). Guidelines for reporting trial protocols and completed trials modified due to the COVID-19 pandemic and other extenuating circumstances: the CONSERVE 2021 statement. JAMA.

[40] Chan AHY, Horne R, Hankins M, Chisari C (2020). The medication adherence report scale: a measurement tool for eliciting patients’ reports of nonadherence. Br J Clin Pharmacol.

[41] Hoffmann TC, Glasziou PP, Boutron I, Milne R, Perera R, Moher D (2014). Better reporting of interventions: template for intervention description and replication (TIDieR) checklist and guide. BMJ..

[42] Alsous M, Alhalaiqa F, Abu Farha R, Abdel Jalil M, McElnay J, Horne R (2017). Reliability and validity of Arabic translation of Medication Adherence Report Scale (MARS) and Beliefs about Medication Questionnaire (BMQ)-specific for use in children and their parents. PLoS One.

[43] Horne R, Weinman J, Hankins M (1999). The beliefs about medicines questionnaire: the development and evaluation of a new method for assessing the cognitive representation of medication. Psychol Health Routledge.

[44] Leon AC, Davis LL, Kraemer HC (2011). The role and interpretation of pilot studies in clinical research. J Psychiatr Res.

[45] Julious SA. Sample size of 12 per group rule of thumb for a pilot study. Pharm Stat. 2005 Cited 2020 Apr 26; Available from: https://scinapse.io/papers/2082494913

[46] Sim J, Lewis M (2012). The size of a pilot study for a clinical trial should be calculated in relation to considerations of precision and efficiency. J Clin Epidemiol.

[47] Sealed Envelope Ltd. Simple randomisation service. 2019. Cited 2020 Apr 26. Available from: https://www.sealedenvelope.com/simple-randomiser/v1/

[48] Rajadhyaksha V (2010). Conducting feasibilities in clinical trials: an investment to ensure a good study. Perspect Clin Res.

[49] Lewis M, Bromley K, Sutton CJ, McCray G, Myers HL, Lancaster GA (2021). Determining sample size for progression criteria for pragmatic pilot RCTs: the hypothesis test strikes back!. Pilot Feasibility Stud.

[50] Al Mazroui NR, Kamal MM, Ghabash NM, Yacout TA, Kole PL, McElnay JC (2009). Influence of pharmaceutical care on health outcomes in patients with type 2 diabetes mellitus. Br J Clin Pharmacol.

[51] Phumipamorn S, Pongwecharak J, Soorapan S, Pattharachayakul S (2008). Effects of the pharmacist’s input on glycaemic control and cardiovascular risks in Muslim diabetes. Prim Care Diabetes.

[52] Ting CY, Ahmad Zaidi Adruce S, Lim CJ, AbdJabar AHA, Ting RS-K, Ting H (2021). Effectiveness of a pharmacist-led structured group-based intervention in improving medication adherence and glycaemic control among type 2 diabetes mellitus patients: a randomized controlled trial. Res Soc Adm Pharm RSAP.

[53] Nelson LA, Greevy RA, Spieker A, Wallston KA, Elasy TA, Kripalani S (2021). Effects of a tailored text messaging intervention among diverse adults with type 2 diabetes: evidence from the 15-month REACH randomized controlled trial. Diab Care.

[54] Bogner HR, Morales KH, de Vries HF, Cappola AR (2012). Integrated management of type 2 diabetes mellitus and depression treatment to improve medication adherence: a randomized controlled trial. Ann Fam Med.

[55] Huang Z, Tan E, Lum E, Sloot P, Boehm BO, Car J (2019). A Smartphone app to improve medication adherence in patients with type 2 diabetes in asia: feasibility randomized controlled trial. JMIR MHealth UHealth.

[56] Cubillos L, Estrada Del Campo Y, Harbi K, Keyserling T, Samuel-Hodge C, Reuland DS (2017). Feasibility and acceptability of a clinic-based mediterranean-style diet intervention to reduce cardiovascular risk for hispanic Americans with type 2 diabetes. Diabetes Educ.

[57] Alessi J, de Oliveira GB, Franco DW, Becker AS, Knijnik CP, Kobe GL (2021). Telehealth strategy to mitigate the negative psychological impact of the COVID-19 pandemic on type 2 diabetes: a randomized controlled trial. Acta Diabetol.

[58] Tam NT, Huy NT, Thoa LTB, Long NP, Trang NTH, Hirayama K (2015). Participants’ understanding of informed consent in clinical trials over three decades: systematic review and meta-analysis. Bull World Health Organ.

[59] Trivedi H, Gray LJ, Seidu S, Davies MJ, Charpentier G, Lindblad U (2017). Self-knowledge of HbA1c in people with type 2 diabetes mellitus and its association with glycaemic control. Prim Care Diabetes.

[60] Maneesakorn S, Robson D, Gournay K, Gray R (2007). An RCT of adherence therapy for people with schizophrenia in Chiang Mai, Thailand. J Clin Nurs.

